# Predictive value of the surgical Apgar score on postoperative complications in advanced gastric cancer patients treated with neoadjuvant chemotherapy followed by radical gastrectomy: a single-center retrospective study

**DOI:** 10.1186/s12893-020-00813-9

**Published:** 2020-07-11

**Authors:** Masato Hayashi, Takaki Yoshikawa, Masahiro Yura, Sho Otsuki, Yukinori Yamagata, Shinji Morita, Hitoshi Katai, Toshirou Nishida

**Affiliations:** Department of Gastric Surgery, National Cancer Center Hospital, 5-1-1 Tsukiji, Chuo-ku, Tokyo, 104-0045 Japan

**Keywords:** Gastrectomy, Neoadjuvant chemotherapy, Surgical Apgar score, Gastric cancer, Postoperative complication

## Abstract

**Background:**

The surgical Apgar score (SAS) or modified SAS (mSAS) has been reported as a simple and easy risk assessment system for predicting postoperative complications in primary surgery for gastric cancer. However, few studies have described the SAS’s utility in gastric surgery after neoadjuvant chemotherapy (NAC).

**Methods:**

One hundred and fifteen patients who received NAC and radical gastrectomy from 2008 and 2015 were included in this study. The SAS was determined by the estimated blood loss (EBL), lowest intraoperative mean arterial pressure, and lowest heart rate. The mSAS was determined by the EBL reassessed using the interquartile values. The predictive values of the SAS/mSAS for postoperative complications were assessed with univariate and multiple logistic regression analyses.

**Results:**

Among the 115 patients, 41 (35.7%) developed postoperative complications. According to analyses with receiver operating characteristic curves of the SAS and mSAS for predicting postoperative complications, the cut-off value of the mSAS was set at 8. The rates of anastomotic leakage, pancreatic fistula, and arrhythmia in patients with high mSAS (> 8) values were higher than in those with low (0–3) and moderate [1–4] mSAS values. A multiple logistic regression analysis showed that the operation time, body mass index, and diabetes mellitus were independent risk factors for postoperative complications. The mSAS was not a significant predictor.

**Conclusion:**

The predictive value of SAS or mSAS for morbidity may be limited in patients who undergo gastric cancer surgery after NAC. Future prospective studies with a large sample size will be needed to confirm the present results.

## Background

Gastrectomy with lymphadenectomy is a core treatment strategy for curing gastric cancer [5]. Although several chemotherapy regimens, such as adjuvant chemotherapy or palliative chemotherapy, have been shown to be effective [6, 7], the prognosis of advanced gastric cancer is still unsatisfactory [1]. In such situations, neoadjuvant chemotherapy (NAC) may be another attractive treatment for improving the prognosis of patients with advanced disease [2–4]; however, surgery after NAC is associated with technical difficulties due to fibrosis induced by chemotherapy, which may cause morbidity. Because surgical complications can not only induce mortality but also decrease a patient’s quality of life, the prediction of morbidity is quite important.

Previously, the surgical Apgar score (SAS) was reported as an easy and simple predictor of postoperative complications in various operations, including gastrectomy [8–11]. SAS is determined by the following intraoperative factors: the estimated blood loss (EBL), lowest intraoperative mean arterial pressure (LMAP), and lowest heart rate (LHR). Furthermore, the modified SAS (mSAS) using a cut-off value of EBL has previously been proposed for assessing the postoperative complication risk in several types of surgery as well [12–15]. However, previous studies investigating the SAS or mSAS only examined gastric cancer patients who received primary surgery with or without adjuvant chemotherapy. Therefore, whether or not the SAS or mSAS are sensitive predictors for gastric cancer patients undergoing surgery after NAC remains unclear.

This study investigated the utility of the SAS and mSAS for predicting morbidity of advanced gastric cancer patients treated with NAC and gastrectomy.

## Methods

### Participants

The medical records of patients at National Cancer Center Hospital, Tokyo, Japan, were retrospectively reviewed to select advanced gastric cancer patients who were treated with NAC and radical gastrectomy from January 2008 to December 2015. The inclusion criteria were [5] gastric adenocarcinoma without previous treatment, [6] a histologically proven diagnosis of adenocarcinoma, and [7] patients who received NAC and radical gastrectomy as a treatment. R1 (Residual Tumor 1) or R2 resection (Residual Tumor 2) cases were excluded from this study. R1/R2 were defined as microscopic/macroscopic residual tumor (positive resection margin or cytology positive), respectively according to the Japanese Gastric Cancer Association Classification [16].

Although NAC has not been established as a standard treatment strategy yet in Japan, the regimens of NAC were basically determined as follows: (1) large type 3 or type 4 gastric cancer was treated according to the protocol of JCOG0501; (2) cT3/cT4 with any N was treated according to the protocol of the in-house study of S-1 and Oxaliplatin; and (3) para-aortic lymph node swelling or extensive nodal swelling along the major branched arteries (bulky-N) was treated according to the protocol of JCOG0405 or JCOG1002.

Following the protocol of JCOG 0405 and JCOG1002, gastrectomy with D2 plus para-aortic nodal dissection was performed. Other trials needed D2 lymphadenectomy. The histological tumor regression of primary tumor was evaluated with the Japanese Classification of Gastric Carcinoma (JCGC) [17].

### The definitions of SAS and mSAS

The SAS was calculated based on the three intraoperative factors of EBL, LMAP, and LHR (Table 1) [8]. The score was calculated from the total points of each category. We also calculated the mSAS in accordance with the method of Miki et al. [12]. In the mSAS, the EBL cut-off value was determined based on its quartile values among the included patients. The median EBL was 519 ml, the 25th percentile was 305 ml, and the 75th percentile was 960 ml (Table 2).

**Table 1:** Surgical Apgar Score (SAS)

	0 point	1 point	2 points	3 points	4 points
Estimated blood loss (mL)	> 1000	601–1000	101–600	≤ 100	–
Lowest mean arterial pressure (mmHg)	< 40	40–54	55–69	≥ 70	–
Lowest heart rate (beats/min)	> 85	76–85	66–75	56–65	≤ 55

Surgical Apgar score was calculated with sum of the points for each category in the course of the procedure

**Table 2:** Modified Surgical Apgar Score (mSAS)

	0 point	1 point	2 points	3 points	4 points
Estimated blood loss (mL)	> 960	519–960	305–518	≤ 304	–
Lowest mean arterial pressure (mmHg)	< 40	40–54	55–69	≥ 70	–
Lowest heart rate (beats/min)	> 85	76–85	66–75	56–65	≤ 55

modified Surgical Apgar Score was calculated with sum of the points as well

### The complication severity evaluation

Postoperative complications were defined as any morbidity occurring within 30 days after surgery. The severity was graded using the Clavien-Dindo Classification [18, 19]. In this study, only complications of grade ≥ IIIa were considered postoperative complications.

### Statistical analyses

The SPSS software program (IBM, Armonk, NY, USA; 25.0 version) was used for all analyses of this study. A nonparametric test (Mann-Whitney U test) was performed for continuous values, whilst Pearson’s chi-squared test or Fisher’s exact test was used for categorical values. A multiple logistic regression analysis was performed to determine the correlation between postoperative complications and perioperative factors in patients who were treated with NAC and radical gastrectomy. Analyses with a receiver operating characteristic (ROC) curve were performed to evaluate the utility of the SAS and mSAS for predicting postoperative complications and to determine the optimum cut-off values of the scores. *P* values of < 0.05 were recognized statistically significant in 2-tailed statistical tests.

## Results

### Postoperative complications

One hundred and twenty patients were treated with NAC followed by radical gastrectomy. Four patients were excluded due to R1 resection, and one patient was excluded due to adenosquamous carcinoma. This study thus ultimately included 115 patients. Among them, 41 patients (35.7%) developed surgical morbidity. As for the incidence of postoperative complications, it was 47 (40.9%). The details of postoperative complications are shown in Table 3.

**Table 3:** Details of Postoperative Complications

Complications	Grade according to Clavien Dindo Classification					Total (%)
	IIIa	IIIb	IVa	IVb	V	
Pancreatic fistula	21	0	0	0	0	21 (18.3%)
Anastomotic leakage	2	2	0	0	0	4 (3.5%)
Abdominal abscess	3	0	0	0	0	3 (2.6%)
Duodenal stump fistula	1	1	0	0	1	3 (2.6%)
Pneumonia	1	0	0	0	0	1 (0.9%)
Bleeding	3	1	0	0	0	4 (3.5%)
Lymph fistula	1	0	0	0	0	1 (0.9%)
Acute kidney failure	0	0	1	0	0	1 (0.9%)
Arrhythmia	0	0	1	0	0	1 (0.9%)
Others	7	1	0	0	0	8 (7.0%)
Total	39	5	2	0	1	47 (40.9%)

### Determination of the cut-off values for predicting postoperative complications

We created the ROC curves of the SAS and mSAS for predicting postoperative complications (data not shown). Because the area under the curve (AUC) of the mSAS was higher than that of the SAS, a further analysis was done using the mSAS. The cut-off value for mSAS was set at 8 based on the curve for predicting complications in this population (AUC of SAS: 0.589, AUC of mSAS: 0.631).

### Clinicopathological features of this population

Table 4 shows the clinicopathological features of this population between low mSAS (< 8) and high mSAS (≥ 8). The body mass index (BMI), operation time, and EBL were significantly greater in the low mSAS group than in the high mSAS group.

**Table 4:** Patients and clinicopathological features between low and high mSAS

Variables	All patients *N* = 115	low mSAS < 8 83 (72.2%)	high mSAS ≥8 32 (27.8%)	*p* value
Age (yr)	63.3 (±10.8)	63.2 (±10.8)	63.8 (±10.9)	0.736
Gender				**0.044**
Male	81 (70.4%)	63 (75.9%)	18 (56.3%)	
Female	34 (29.6%)	20 (24.1%)	14 (43.8%)	
Body Mass Index (BMI)	22.3 (±3.3)	22.7 (±3.6)	21.1 (±2.8)	**0.027**
BMI ≥ 22	60 (52.2%)	46 (55.4%)	14 (43.8%)	**0.001**
Diabetes Mellitus	9 (7.8%)	7 (8.4%)	2 (6.3%)	1
Smoking history	73 (63.5%)	58 (69.9%)	15 (46.9%)	**0.030**
American Society of Anesthesiologists Physical Status (ASA-PS)				0.703
1	11 (9.6%)	7 (8.4%)	4 (12.5%)	
2	94 (81.7%)	68 (81.9%)	26 (81.3%)	
3	10 (8.7%)	8 (9.6)	2 (6.3%)	
Tumor location^a^				0.097
Upper	46 (40%)	38 (45.8%)	8 (25.0%)	
Middle	39 (33.9%)	26 (31.3%)	13 (40.6%)	
Lower	20 (17.4%)	11 (13.3%)	9 (28.1%)	
Whole	10 (8.7%)	8 (9.6%)	2 (6.3%)	
ycStage				0.302
I	3 (2.6%)	3 (3.6%)	0	
II	47 (40.9%)	31 (37.3%)	16 (50.0%)	
III	65 (56.5%)	49 (59.0%)	16 (50.0%)	
Neoadjuvant chemotherapy regimen				0.057
S-1^b^ and cisplatin	74 (64.3%)	51 (61.4%)	23 (71.9%)	
S-1^b^ and oxaliplatin	14 (12.2%)	8 (9.6%)	6 (18.8%)	
S-1^b^, docetaxel and cisplatin	13 (11.3%)	10 (12.0%)	3 (9.4%)	
Others	14 (12.2%)	14 (16.9%)	0	
Surgical Procedure				**0.001**
Distal gastrectomy	29 (25.2%)	13 (15.7%)	16 (50.0%)	
Total gastrectomy	83 (72.3%)	68 (81.9%)	15 (46.9%)	
Other	3 (2.6%)	2 (2.4%)	1 (3.1%)	
Operation time (min)	337.1 (±111.1)	365.6 (±113.5)	263.4 (±59.8)	**< 0.001**
Surgical Apgar score	6.25 (±1.1)	5.77 (±1.2)	7.50 (±0.67)	**< 0.001**
modified Surgical Apgar Score	6.34 (±1.5)	5.65 (±1.2)	8.16 (±0.46)	**< 0.001**
Estimated blood loss (ml)	711.5 (±627.0)	898.1 (±644.3)	227.6 (±112.9)	**< 0.001**
Lowest mean arterial pressure	55.6 (±6.6)	54.8 (±6.5)	57.6 (±6.4)	**0.034**
Lowest heart rate	58.7 (±8.4)	59.8 (±8.8)	56.0 (±6.7)	**0.034**
Extent of lymphadenectomy				**0.023**
D2^c^	48 (41.7%)	31 (37.3%)	17 (53.1%)	
D2 + ^d^	40 (34.8%)	27 (32.5%)	13 (40.6%)	
D3^e^	27 (23.5%)	25 (30.1%)	2 (6.3%)	
ypT^a^ factor				0.346
0	4 (3.5%)	3 (3.6%)	1 (3.1%)	
T1a	1 (0.9%)	1 (1.2%)	0	
T1b	13 (11.3%)	10 (12.0%)	3 (9.4%)	
T2	17 (14.8%)	15 (18.1%)	10 (31.3%)	
T3	51 (44.3%)	36 (43.4%)	15 (46.9%)	
T4a	25 (21.7%)	15 (18.1%)	10 (31.3%)	
T4b	4 (3.5%)	3 (3.6%)	1 (3.1%)	
ypN^a^ factor				0.781
0	39 (33.9%)	30 (36.1%)	9 (28.1%)	
1	24 (20.9%)	18 (21.7%)	6 (18.8%)	
2	26 (22.6%)	19 (22.9%)	7 (21.9%)	
3	26 (22.6%)	16 (19.3%)	10 (31.3%)	
M factor				0.725
0	104 (90.4%)	74 (89.2%)	30 (93.8%)	
1	11 (9.6%)	9 (10.8%)	2 (6.3%)	
ypStage^a^				0.392
0	3 (2.6%)	3 (3.6%)	0	
I	19 (16.5%)	16 (19.3%)	3 (9.4%)	
II	37 (32.3%)	25 (30.1%)	12 (37.5%)	
III	45 (39.1%)	30 (36.1%)	15 (46.9%)	
IV	11 (9.6%)	9 (10.8%)	2 (6.3%)	
postoperative complication^f^ ≥ IIIa	41 (35.7%)	35 (42.2%)	6 (18.8%)	**0.029**
Histological response of primary lesion				0.673
0	2 (1.7%)	2 (2.4%)	0	
1a	33 (28.7%)	24 (28.9%)	9 (28.1%)	
1b	30 (26.1%)	22 (26.5%)	8 (25.0%)	
2	47 (40.9%)	32 (38.6%)	15 (46.9%)	
3	3 (2.6%)	3 (3.6%)	0	

^a^According to the seventh edition of the International Union Against Cancer tumor, node, metastasis (TNM) classification system

^b^Tegafur/Gimeracil/Oteracil: Product name is TS-1

^c^D2 lymphadenectomy: Dissection filed for distal gastrectomy: #1, #3a, #3b, #4sb, #4d, #5, #6, #7, #8a, #9, #11p, #12aDissection filed for total gastrectomy: #1, #2, #3a, #3b, #4sa, #4sb, #4d, #5, #6, #7, #8a, #9, #10, #11p, #11d, #12a

^d^D2+ lymphadenectomy: D2 plus either #16a2/#16b1

^e^D3 lymphadenectomy: D2 plus both #16a2 and #16b1

^f^According to Clavien-Dindo Classification

Table 5 shows the risk stratification of complications according to the mSAS, which was divided into 3 categories: low mSAS, 0–3; moderate mSAS, 4–7; high mSAS, 8–10. The risk of complication in the high mSAS group was significantly lower than that in the low mSAS group. Table 6 shows that the rates of pancreatic fistula, anastomotic leakage, and arrhythmia were significantly higher in the low mSAS group than in the moderate and high mSAS groups.

**Table 5:** Risk stratification of complication according to mSAS

	with complications^a^ *N* = 41 (35.7%)	without complications^a^ *N* = 74 (64.3%)	OR (95% CI)	*p* value
mSAS 0–3	3 (7.3%)	1 (1.4%)	1	
mSAS 4–7	32 (78.0%)	47 (63.5%)	0.23 (0.023–2.280)	0.208
mSAS 8–10	6 (14.6%)	26 (35.1%)	0.08 (0.007–0.875)	**0.039**

^a^Grade IIIa or higher According to Clavien-Dindo Classification

**Table 6:** Comparison of complication^a^ among categorized mSAS

	mSAS 0–3 *N* = 4	mSAS 4–7 *N* = 79	mSAS 8–10 *N* = 32	*p* value
Pancreatic fistula	2 (50.0%)	17 (21.5%)	2 (6.3%)	0.042
anastomotic leakage	1 (25.0%)	3 (3.8%)	0 (0%)	0.035
arrhythmia	1 (25.0%)	0 (0%)	0 (0%)	< 0.001

^a^Grade IIIa or higher According to Clavien-Dindo Classification

### Results of a logistic regression analysis of the factors associated with postoperative complications

Table 7 shows the results of univariate and multiple logistic regression analyses for postoperative complications. The univariate analysis revealed significant differences in the gender, BMI, diabetes mellitus (DM), smoking history, ycStage, operation time, EBL and mSAS. Except for EBL, which was excluded because it was an apparent confounding factor for the mSAS, these factors were included as covariates in a multiple logistic regression analysis of factors predicting complications. In the multiple logistic regression analysis, the BMI, presence of DM, and operation time were detected as independent risk factors for postoperative complications. The mSAS was not identified as a risk factor.

**Table 7:** Univariate analysis and multiple logistic regression analysis for postoperative complications

	Univariate analysis			Multiple logistic regression analysis		
Variables	with complications^b^ *N* = 41 (35.7%)	without complications^b^ *N* = 74 (64.3%)	*p* value	OR	95% CI	*p* value
Gender			**0.034**	0.85	0.24–3.02	0.82
Male	34 (82.9%)	47 (63.5%)				
Female	7 (17.1%)	27 (36.5%)				
Body Mass Index (BMI)	24.4 (±3.2)	21.1 (±2.7)	**< 0.001**	3.74	1.33–10.51	**0.012**
Diabetes Mellitus	7 (17.1%)	2 (2.7%)	**0.010**	13.5	2.00–91.0	**0.008**
Smoking history	32 (78.0%)	41 (55.4%)	**0.017**	2.39	0.74–7.70	0.145
ycStage^a^			**0.020**	1.69	0.62–4.57	0.303
I	0 (0%)	3 (4.1%)				
II	11 (26.8%)	36 (48.6%)				
III	30 (73.2%)	35 (47.3%)				
Operation time (min)	395.7 (±138.3)	304.7 (±76.2)	**< 0.001**	4.07	1.36–12.12	**0.012**
mSAS	5.88 (±1.6)	6.61 (±1.4)	**0.018**	0.9	0.63–1.27	0.54
Estimated blood loss (ml)	899.7 (±788.1)	607.2 (±492.7)	**0.011**	–	–	–
Lowest mean arterial pressure	56.2 (±6.4)	55.3 (±6.7)	0.057	–	–	–
Lowest heart rate	60.7 (±8.2)	57.6 (±8.4)	0.492	–	–	–

^a^According to the seventh edition of the International Union Against Cancer tumor, node, metastasis (TNM) classification system

^b^Grade IIIa or higher According to Clavien-Dindo Classification

### Results of an analysis of confounding factors for the mSAS

Confounding factors of surgical outcome and mSAS were considered. An analysis of the confounding factors revealed that the low mSAS group included significantly greater proportions of patients with a high BMI and long operation time than the high mSAS group (Table 4).

## Discussion

Before obtaining the results of our study, SAS and mSAS have been considered predictors for surgical morbidity in patients who receive surgery for gastric cancer. However, whether or not this remains true in patients who receive neoadjuvant chemotherapy followed by surgery is unclear. Unexpectedly, this study failed to demonstrate the utility of the SAS or mSAS for predicting postoperative complications in this patient population. The mSAS was not an independent predictor of morbidity. To our knowledge, this study is the first report to evaluate the correlation between the SAS and complications in patients treated with NAC followed by radical gastrectomy.

Several previous studies have demonstrated the utility of SAS for predicting complications [9, 10], although other studies have described the usefulness of the mSAS but not the SAS [12, 14]. The utility of the SAS seems to depend on the type of surgery or the characteristics of the cohort. We considered the reasons why the SAS and mSAS were not found to be risk factors for complications in the present study. In our study, a high BMI, the presence of DM, and a long operation time were identified as independent risk factors for postoperative complications. In the low mSAS group, the proportions of patients with a high BMI and long operation time were higher than in the high mSAS group, suggesting that these factors were confounders of the mSAS. These results suggested that patients with a high BMI or longer operation time easily develop surgical morbidities and that the predictive value of the mSAS was inferior to that of a high BMI or long operation time.

Anesthesia may also have influenced the results. The LHR and LMAP, which are included in the SAS and mSAS, can easily be affected by anesthesia. Deep anesthesia can reduce the arterial pressure without any bleeding, and the use of high doses of opioids can prevent an increase in the heart rate caused by bleeding or dehydration. Ideally, the mSAS should be evaluated under the same conditions of anesthesia. Unfortunately, however, anesthesia in our institution was managed by several doctors under different policies during the study period. The lack of statistical significance of the SAS and mSAS for predicting complications might therefore have been influenced by these different management approaches.

This study and Miki’s [12] study differ in some respects, although both studies investigated the predictive value of the mSAS in gastric cancer patients. First, the EBL of our population was much higher than that in Miki’s study, possibly due to surgical difficulties, such as fibrosis induced by NAC or extensive lymphadenectomy, which was selected in more than half of the present cohort. These differences might have caused our results to differ from those of Miki’s study. Second, our cut-off value of mSAS was much higher than that in the previous report, even though our EBL was higher, possibly due to the very high incidence of complications in the present population. This point might be another reason for the differences between the present and previous study.

In this study, a high BMI, the presence of DM, and a long operation time were detected as risk predictors for postoperative complications in patients treated with NAC. These factors are well known predictors of complications in primary surgery [20–23]. A high BMI is closely related to excessive visceral fat, which may extend the operation time and impair lymph node dissection [24, 25]. Thus, a high BMI increases the difficulty of the whole operation. Excessive visceral fat easily induces metabolic syndrome, including DM, which makes patients more susceptible to infection and can inhibit wound healing. A long operation time has been reported to be a risk factor of postoperative complications, accelerating the speed of body metabolism and increasing the consumption of nutrition [22]. These factors would not be changed after NAC.

Several limitations associated with the present study warrant mention. First, this was a retrospective, single-center study. Although our study population was mostly limited to patients enrolled in prospective clinical trials, the possibility of several biases could not be completely excluded. Furthermore, we cannot deny the possibility of type 2 error in this study because of the small sample size. For these reasons, a prospective study with a large sample size is needed to confirm our results. Second, the sample size was small, so the predictive value of the mSAS might have been underestimated in this study.

## Conclusion

The predictive value of SAS or mSAS for morbidity may be limited in patients who undergo gastric cancer surgery after NAC. A prospective study with a large sample size will be needed to confirm the present results.

## Data Availability

The datasets used and analyzed during the current study are available from the corresponding author on reasonable request.

## Abbreviations

SAS: Surgical Apgar score
mSAS: modified SAS
NAC: Neoadjuvant chemotherapy
EBL: Estimated blood loss
LMAP: Lowest mean arterial pressure
LHR: Lowest heart rate
ROC: Receiver operating characteristics
JCGC: Japanese Classification of Gastric Carcinoma
DM: Diabetes mellitus
BMI: Body mass index
ASA-PS: American Society of Anesthesiologists Performance Status
